# Does curcumin have an effect on sleep duration in metabolic syndrome patients? 

**Published:** 2021

**Authors:** Maryam Saberi-Karimian, Hamideh Ghazizadeh, Elham Mohammadzadeh, Gordon A. Ferns, Majid Ghayour-Mobarhan, Amirhosein Sahebkar

**Affiliations:** 1 *International UNESCO center for Health Related Basic Sciences and Human Nutrition, Mashhad University of Medical Sciences, Mashhad, Iran*; 2 *Brighton & Sussex Medical School, Division of Medical Education, Falmer, Brighton, Sussex BN1 9PH, UK*; 3 *Cardiovascular Research Center, Faculty of Medicine, Mashhad University of Medical Sciences, Mashhad, Iran*; 4 *Biotechnology Research Center, Mashhad University of Medical Sciences, Mashhad, Iran*; 5 *Student Research Committee, Mashhad University of Medical Sciences, Mashhad, Iran*; 6 *Metabolic Syndrome Research Center, Faculty of Medicine, Mashhad University of Medical Sciences, Mashhad, Iran*

**Keywords:** Curcumin, Sleep duration, Metabolic syndrome

## Abstract

**Objective:**

Sleep-duration is related to obesity. Curcumin can affect behavioral changes that arise from sleep deprivation in animal models. In this study, we assessed the effects of curcumin on sleep-duration in metabolic-syndrome (MetS) patients.

**Materials and Methods:**

This study was a double-blind clinical trial in 120 adults with MetS. All participants received crude curcuminoids in a simple formulation (n=40), phospholipidated curcuminoids (n=40) or placebo (n=40) 1 g/day during 6 weeks. Demographic data, anthropometric indices and serum biochemical factors were documented for all volunteers at baseline and after the intervention. A standard questionnaire was used for evaluating physical-activity-level (PAL) and patients’ sleep-duration, including night time sleep and daily napping. Based on the time of sleep, sleeping hours were classified into: night time sleep; daily naps and total sleeping hours in 24 hours.

**Results:**

A total of 120 participants aged 38.72±10.05 years old were enrolled into the study. We did not find significant differences in biochemical factors, sleep-duration or PAL at baseline among the 3 groups (p>0·05). Moreover, curcumin did not exert any significant effect on sleep-duration before, or after, adjustment for confounding factors in the overweight and obese individuals, or in total population (p>0.05).

**Conclusion:**

The results showed that curcumin does not have an effect on sleep-duration in subject with MetS.

## Introduction

Metabolic syndrome (MetS) is a multifaceted disorder with a cluster of cardiovascular disease (CVD) risk factors including abdominal obesity, hyperglycemia, hypertriglyceridemia, hypertension and decreased high-density lipoprotein cholesterol (HDL-C) concentrations (Reaven, 1988 ▶). The prevalence of MetS has increased globally due to excessive consumption of energy-dense foods a long with sedentary lifestyle. A high prevalence of MetS in Iranian adults has been reported (36.9 and 34.6% respectively based on the Adult Treatment Panel III criteria (ATP III) and the International Diabetes Federation (IDF) criteria). Moreover, the prevalence of MetS was reported to be higher in women compared with men (Amirkalali et al., 2015 ▶). Furthermore 6.5% of the Iranian people, and 45% of obese girls have MetS in Mashhad, Khorasan Razavi, Iran (Mirhosseini et al., 2009 ▶). 

There is a need for adjunctive, or alternative therapies, to reduce the residual CVD risk that remains following taking statin in these individuals, and a lack of tolerability or therapeutic response in certain groups of patients (Mohammadi et al., 2013 ▶). It has been suggested that phytochemicals may exert some metabolic and health advantages (Karalis, 2008 ▶; Miquel et al., 2002 ▶; Panahi et al., 2014a ▶; Panahi et al., 2014b ▶; Sahoo et al., 2008 ▶; Visioli and Davalos, 2011 ▶; Sahebkar, 2013 ▶;). Turmeric is used for the treatment of hepatic disorders, diabetic wounds, rheumatism, biliary disorders, anorexia, and cough (Panahi et al., 2012 ▶; Sahebkar, 2011 ▶). Curcuminoids are known as the main active components of turmeric , which comprise curcumin (diferuloylmethane), demethoxycurcumin and bisdemethoxycurcumin (Sahoo et al., 2008 ▶). It has been reported that curcuminoids possess a multitude of biological (Franco-Robles et al., 2013 ▶; Marshall et al., 2008 ▶; Sahebkar et al., 2013 ▶; Taheri 2006 ▶; Taheri et al., 2004 ▶) and pharmacological effects (Kumar and Singh, 2008 ▶; Noorafshan et al., 2017a ▶; Riemann et al., 2007 ▶; Saberi-Karimian et al., 2018 ▶; Zhang et al., 2013 ▶) and can be used to treat a variety of human disorders. 

Some studies have reported a link between obesity and sleep duration; for example, short sleep duration was shown to be a risk factor for the development of obesity. Several molecular mechanisms have been suggested to explain this effect (Shi et al., 2008 ▶). Energy intake and expenditure as well as physical activity level can be affected by sleep duration (Taheri et al., 2004 ▶; Taheri, 2006 ▶). Other studies have shown contradictory results (Marshall et al., 2008 ▶). Inadequate sleep can reduce the volume of hippocampus (Riemann et al., 2007 ▶). It is suggested that sleep deprivation may increase the mediators of oxidative stress in some brain areas (Zhang et al., 2013 ▶). In rats’ hippocampus, curcumin has been shown to inhibit the structural and behavioral changes under these conditions (Noorafshan et al., 2017a ▶). Moreover, curcumin can limit oxidative damage and behavioral changes due to sleep deprivation in mice (Kumar and Singh, 2008 ▶).

It has been reported that short sleep duration is related to overweight and augmented blood pressure in adolescents in Korea, although there was no association with MetS (Lee and Park, 2014 ▶). Here, we evaluated the curcumin’s effects on sleep duration in subjects with MetS.

## Materials and Methods

**Study design**

**Figure 1:**
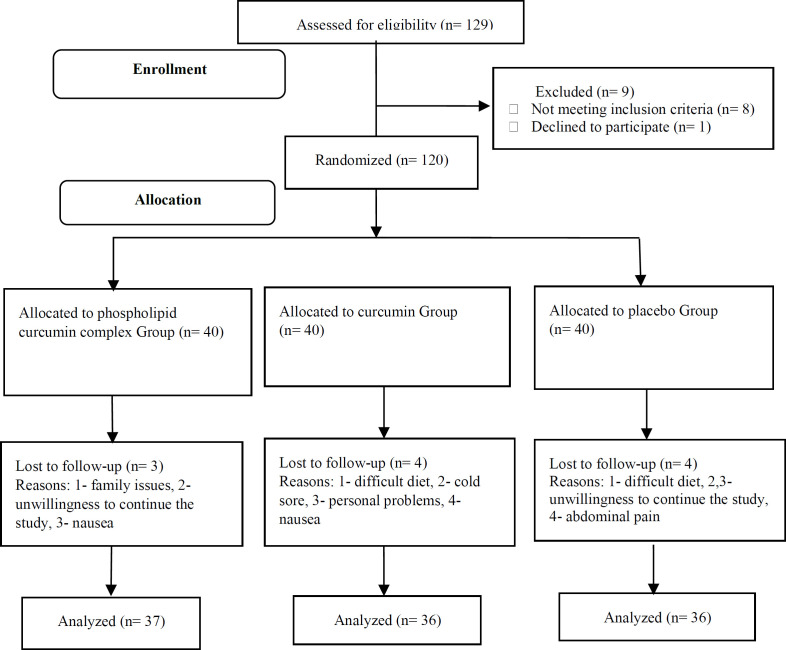
The flow chart of the study design

A total of 120 participants (aged 18 to 65 years) with metabolic syndrome were enrolled into this clinical trial. The subjects were blinded to the study groups and were randomly allocated to: Group 1 and 2- the curcumin groups taking curcumin capsules in simple (1 g/day; n=40) or modified formula (1 g/day=200 mg pure curcumin/day; n=40), and Group 3- control group taking a placebo (lactose & starch; n=40) during 6 weeks. The flow chart of the study design has been shown in Figure 1. 

Random number tables were used to randomize patients to curcumin and placebo groups (Fleiss, 2011 ▶). Inclusion and exclusion criteria were as previously detailed (Lee and Park, 2014 ▶). The inclusion criteria were age of 18 to 65 years with a history of MetS based on the IDF guidelines (2010). IDF criteria are waist circumference more than 94 and 80 (cm) in male and female, respectively, plus any 2 of the following criteria: 1) triglyceride equal or above 150 mg/dl or taking drug for this kind of dyslipidemia. 2) HDL-C less than 40 and 50 mg/dl respectively in males and females or specific treatment for this lipid abnormality. 3) Systolic blood pressure equal or above 130 or diastolic blood pressure) equal or above 85 mmHg or treatment of previously diagnosed hypertension. 4) Fasting plasma glucose equal or above 100 mg/dl or previously diagnosed type 2 diabetes.

Women who were breastfeeding, or pregnant, or individuals with systemic diseases, or those taking any drugs and nutritional supplements during the project period, were excluded.

The current study was a sub-study from another work registered in the Iranian Registry of Clinical Trials (IRCT2014052014521N3) (Saberi-Karimian et al., 2018 ▶). The sample size was calculated 35 individuals per group (considering α=0.05 and β=0.02) in the original work based on the serum triglycerides changes levels according to our previous project (Mohammadi et al., 2013 ▶). 

Demographic data, blood pressure and anthropometric indices were determined at baseline and after 6 weeks. Blood samples were collected after a 12-hour fasting. Complete blood count (CBC) and serum biochemical factors were determined using routine methods. 

**Physical activity level (PAL)** **and sleep duration**

PAL was calculated using the questionnaire devised by Vasconcellos and Anjos (2003) ▶. The subjects were divided into 5 categories based on human energy requirement involved: a) extremely inactive (less than 1.40), b) sedentary (1.40 to 1.69), c) moderately active (1.70 to 1.99), d) vigorously active (2.00 to 2.40), or e) extremely active (more than 2.40) (James and Schofield 1990). 

We used a standard questionnaire (self-declaration form) to assess patients’ sleep duration, including nighttime sleep and daily nap (James and Schofield 1990). According to the time of sleep, sleeping hours were classified into: a. nighttime sleep (sleep duration only in night); b. daily naps; c. total sleeping hours in 24 hours (including nighttime sleep duration plus the duration of daily naps). 

**Statistical analysis**

Data are stated as mean±SD (for normally distributed variables) or median (first and third quartiles) (for non-normally distributed data). We determined the normality of data using the Kolmogorov-Smirnov test. According to data distribution pattern, we used Student's t-test, Analysis Of Variance (ANOVA), Mann-Whitney U, and Kruskal-Wallis tests to compare data among the groups. We used a general linear model to detect the independent association between sleep duration and physical activity with Curcumin supplementation. A p-value (two-tailed) less than 0.05 was considered statistically significant using SPSS version 16.0 (SPSS Inc. Chicago, IL, USA).

## Results

In this study, a population of 120 subjects including 71.1% male and 28.3% female (aged 38.72±10.05 years) was recruited. 

**Clinical characteristics**

The clinical characteristics of the population at baseline are reported in Table 1. Serum biochemical variables, sleep duration and physical activity at baseline did not show a difference among the three study groups (p>0·05).

Changes of clinical and baseline characteristics of the population between baseline and after 6 weeks of intervention in the three study groups are summarized in Table 2. There was no significant difference in any of the variables such as sleep, PAL and BMI between before and after the intervention (Table 2). 

**Table 1:** Clinical and biochemical features in subjects at baseline

**Variables**			**Curcumin-phospholipid complex (N=40)**	**Curcumin (N=40)**	**Placebo (N=40)**	**p-value**
**Age (years)**			40.05±10.48	37.52±9.47	38.59±10.28	0.92
**BMI (kg/m** ^2^ **)**			30.66±5.06	30.67±3.57	31.22±4.67	0.53
**Sex**	Female% (n)		62.5 (25)	77.5 (31)	75.0 (30)	0.280
	Male% (n)		37.5 (15)	22.5 (9)	25.0 (10)	
**FAT%**			34.51±8.07	35.42±6.12	35.21±7.86	0.86
**Total sleep (hr)**			5.81±0.93	5.69±1.02	5.36±1.02	0.56
**Nightly sleep (hr)**			4.75±0.79	5.05±0.91	4.76±0.95.374	0.41
**Daily nap (hr)**			1.00±1.10	0.62±0.74	0.67±0.88	0.44
**Physical activity level (PAL); n(%)**		Extremely inactive	28 (77.8)	29 (80.6)	33 (84.6)	0.704
		Sedentary	6 (16.7)	5 (13.9)	5 (12.8)	
		Moderately active	-	-	-	
		Vigorously active	-	-	-	
		Extremely active	2 (5.6)	2 (5.6)	1 (2.6)	

Values are expressed as mean±SD for normally distributed data, and median and interquartile range for non-normally distributed data. Between groups comparisons were made by parametric statistical analysis for normally distributed data and nonparametric test for non-normally distributed data. BMI, body mass index.

**Table 2:** The effects of curcumin on PAL, nightly sleep, BMI and daily nap in subjects with metabolic syndrome

**Groups**		**Curcumin-phospholipid complex (N=40)**	**Curcumin (N=40)**	**Placebo (N=40)**	**p-value**
**Physical activity level (PAL)**	Before	1.23 (0.28)	1.22 (0.25)	1.23 (0.21)	0.72
	After	1.51 (0.31)	1.57 (0.37)	1.54 (0.31)	0.83
	Changes at baseline and after 6 weeks intervention	0.28 (0.14)	0.30 (0.13)	0.29 (0.17)	0.32
**BMI (kg/m** ^2^ **)**	Before	30.66 (5.06)	30.67 (3.57)	31.22±4.67	0.54
	After			31.30±4.78	0.56
	Changes at baseline and after 6 weeks intervention	-0.19 (0.68)	-0.30 (0.76)	-0.10±0.77	0.37
**Nighty sleep (hr)**	Before	4.75±0.79	5.05±0.91	4.76±0.94	0.41
	After	4.17±1.72	3.87±2.01	3.70±1.98	0.38
	Changes at baseline and after 6 weeks intervention	-0.48±1.42	-1.07±2.25	-0.97±1.96	0.63
**Daily nap (hr)**	Before	1.00±1.10	0.62±0.74	0.67±0.88	0.44
	After	0.67±0.79	0.60±0.70	0.55±0.78	0.55
	Changes at baseline and after 6 weeks intervention	-0.32±0.94	-0.02±0.42	-0.12±0.60	0.71
**Total sleep (hr)**	Before	5.81±0.93	5.69±1.02	5.36±1.02	0.05
	After	4.85±1.98	4.47±2.38	4.25±2.28	0.33
	Changes at baseline and after 6 weeks intervention	-0.86±1.93	-1.10±2.44	-1.05±2.37	0.88

Values are expressed as mean±SD for normally distributed data, and median and interquartile range for non-normally distributed data. Between groups comparisons were made by parametric statistical analysis for normally distributed data and nonparametric test for non-normally distributed data.

**Table 3:** The effects of curcumin on sleep duration in subjects with metabolic syndrome

**Groups**	**Nighty sleep (hr)**		**B**	**Confidence interval 95%**	**p-value**
**BMI<30 (kg/m** ^2^ **)**	Changes at baseline and after 6 weeks intervention	Curcumin-phospholipid complex (N=40)	0.72	-2.06 to 3.51	0.58
		Curcumin (N=40)	-3.08	-7.13 to 0.95	0.12
		Placebo (N=40)	Ref.1		
**BMI≥30(kg/m** ^2^ **)**	Changes at baseline and after 6 weeks intervention	Curcumin-phospholipid complex (N=40)	0.23	-0.36 to 0.83	0.44
		Curcumin (N=40)	-0.35	-0.95 to 0.25	0.24
		Placebo (N=40)	Ref.1		
	Daily nap (hr)				
**BMI<30 (kg/m** ^2^ **)**	Changes at baseline and after 6 weeks intervention	Curcumin-phospholipid complex (N=40)	-0.45	-1.17 to 0.27	0.20
		Curcumin (N=40)	0.19	-0.84 to 1.24	0.69
		Placebo (N=40)	Ref.1		
**BMI≥30 (kg/m** ^2^ **)**	Changes at baseline and after 6 weeks intervention	Curcumin-phospholipid complex (N=40)	-0.18	-0.44 to 0.04	0.13
		Curcumin (N=40)	-0.19	-0.43 to 0.06	0.11
		Placebo (N=40)	Ref.1		
	Total sleep (hr)				
**BMI<30 (kg/m** ^2^ **)**	Changes at baseline and after 6 weeks intervention	Curcumin-phospholipid complex (N=40)	0.27	-2.89 to 3.44	0.85
		Curcumin (N=40)	-2.88	-7.48 to 1.70	0.20
		Placebo (N=40)	Ref.1		
**BMI≥30 (kg/m** ^2^ **)**	Changes at baseline and after 6 weeks intervention	Curcumin-phospholipid complex (N=40)	0.04	-0.69 to 0.78	0.90
		Curcumin (N=40)	-0.55	-1.29 to 0.18	0.14
		Placebo (N=40)	Ref.1		

General Linear Model was used after adjusting for changes in the neutrophils, and serum lipoprotein (a) and adiponectin (26) at baseline and after 6 weeks intervention. Values are expressed as mean±SD for normally distributed data. Between groups comparisons were made by parametric statistical analysis for normally distributed data. BMI; body mass index.

**Table 4:** Association between curcumin treatment with sleep duration

**Changes in variable at baseline and after 6 weeks intervention**		**B**	**Confidence interval 95%**	**p-value**
**Daily nap (hr)**	Curcumin-phospholipid complex (N=40)	-0.24	-0.56 to 0.08	0.13
	Curcumin (N=40)	-0.13	-0.49 to 0.23	0.47
	Placebo (N=40)	Ref.1		
**Nighty sleep (hr)**	Curcumin-phospholipid complex (N=40)	0.25	-0.72 to 1.22	0.60
	Curcumin (N=40)	-1.08	-2.18 to 0.01	0.05
	Placebo (N=40)	Ref.1		
**Total sleep (hr)**	Curcumin-phospholipid complex (N=40)	-0.07	-1.20 to 1.05	0.89
	Curcumin (N=40)	-1.35	-2.62 to -0.08	0.03
	Placebo (N=40)	Ref.1		

General Linear Model was used after adjusting for, job, marital status, the number of family members, the head of the family, changes in the neutrophils, and serum lipoprotein (a) and adiponectin (26) at baseline and after 6 weeks intervention. Values are expressed as mean±SD for normally distributed data, and median and interquartile range for non-normally distributed data. Between groups comparisons were made by parametric statistical analysis for normally distributed data and nonparametric test for non-normally distributed data. BMI; body mass index.

**Table 5:** Correlation between sleep duration and PAB

**Variable**		**PAB before (HK)**	**Changes in PAB at baseline and after 6 weeks intervention (HK)**
Daily nap before (hr)	Spearmans rho	0.16	
	p-value	0.09	
Changes in daily nap at baseline and after 6 weeks intervention (hr)	Spearmans rho		-0.02
	p-value		0.83
Nighty sleep before (hr)	Spearmans rho	-0.86	
	p-value	0.38	
Changes in nighty sleep at baseline and after 6 weeks intervention (hr)	Spearmans rho		-0.11
	p-value		0.06
Total sleep before (hr)	Spearmans rho	0.05	
	p-value	0.58	
Changes in total sleep at baseline and after 6 weeks intervention (hr)	Spearmans rho		-0.11
	p-value		0.23

PAB, prooxidant-antioxidant balance.

According to Tables 3 and 4, curcumin did not have significant effects on sleep duration (before and after adjusting for confounding factors) in overweight and obese subjects and total population, respectively (p>0.05).

As summarized in Table 5, there was no association between sleep duration and prooxidant-antioxidant balance before and after the intervention.

## Discussion

To the best of our knowledge, this was the first clinical trial testing whether curcumin supplementation can affect sleep duration in subjects with MetS. One of the most important results was that all subjects suffered from short sleep duration (Table 1). An explanation for this finding can be obesity, because all subjects had MetS and their BMI were >30 kg/m^2^. Several molecular mechanisms have been suggested to explain this observation (Shi et al., 2008 ▶). The short sleep duration can change the energy intake and expenditure balance (Spiegal et al., 1999 ▶), metabolic and endocrine function, leptin and ghrelin concentrations (Taheri et al., 2004 ▶), the adipocyte circadian clock (Shi et al., 2008 ▶), as well as the levels of insulin, cortisol, growth hormone and interleukin-6 that could contribute to metabolic dysfunction (Vgontzas et al., 2004 ▶; Taheri, 2006 ▶; Van Cauter et al., 1991 ▶).

In addition, our findings indicated that sleep duration was not affected by curcumin supplementation after 6 weeks of supplementation in subjects with MetS. It has been shown that autonomic, neuroendocrine and immune system homeostasis can be regulated by sleep (McEwen, 2006 ▶; Moreira, 2006 ▶; Steiger, 2007 ▶). Insufficient sleep can disturb mental health and psychological balance. Poor sleep quality may also cause weight loss (McEwen, 2006 ▶; Cirelli, 2006 ▶; ), and behavioral changes (Obermeyer et al., 1991 ▶).

There is limited data on the effects of supplementation with curcuminoids and sleep quality in man. However, there are some related animal studies on sleep deprivation (Kumar and Singh, 2008 ▶; Noorafshan et al., 2017b ▶; Pezze et al., 2016 ▶; Noorafshan et al., 2017b ▶). 

 Kumar and Singh (2008) ▶ assessed the molecular mechanism of curcumin’s effects in improving sleep deprivation in male mice. Their results suggested that the curcumin’s positive effect on sleep behavioral alterations and oxidative damage was caused by the modulation of nitric oxide (Kumar and Singh, 2008 ▶). In rats, sleep deprivation can cause structural changes in the medial prefrontal cortex and produce memory impairment (Noorafshan et al., 2017b ▶). The medial prefrontal cortex take vital innervations from the hippocampus and the brain stem (Pezze et al., 2016 ▶). Another animal study reported that curcumin can protect this area (Noorafshan et al., 2017b ▶). 

There is a relationship between sleep duration and prooxidant-antioxidant balance (Kumar and Singh, 2007 ▶). Sleep deprivation can augment the generation of free radicals and attenuation of antioxidative defense. We evaluated the curcumin's effects on prooxidant-antioxidant balance in a previous study (Ghazimoradi et al., 2017 ▶). Our results did not show any effects in MetS patients. Moreover, in the current study, there was no association between sleep duration and prooxidant-antioxidant balance at baseline and after 6 weeks of supplementation. The results of this study do not support any significant effects of curcumin on sleep. 

This was a sub-study of our previous double-blinded controlled trial on curcumin’s effects on CVD risk factors in subjects with MetS. It may have been better to assess sleep quality along with sleep duration using actigraphy for measuring sleep duration as a gold standard. 

The results showed that sleep-duration was not affected by curcumin supplementation in MetS patients. 
